# Binding and neutralization of vascular endothelial growth factor (VEGF) and related ligands by VEGF Trap, ranibizumab and bevacizumab

**DOI:** 10.1007/s10456-011-9249-6

**Published:** 2012-02-03

**Authors:** Nicholas Papadopoulos, Joel Martin, Qin Ruan, Ashique Rafique, Michael P. Rosconi, Ergang Shi, Erica A. Pyles, George D. Yancopoulos, Neil Stahl, Stanley J. Wiegand

**Affiliations:** Regeneron Pharmaceuticals Inc., 777 Old Saw Mill River Road, Tarrytown, NY 10591 USA

**Keywords:** VEGF receptor, Aflibercept, Affinity, Age-related macular degeneration, Placental growth factor

## Abstract

Pharmacological inhibition of VEGF-A has proven to be effective in inhibiting angiogenesis and vascular leak associated with cancers and various eye diseases. However, little information is currently available on the binding kinetics and relative biological activity of various VEGF inhibitors. Therefore, we have evaluated the binding kinetics of two anti-VEGF antibodies, ranibizumab and bevacizumab, and VEGF Trap (also known as aflibercept), a novel type of soluble decoy receptor, with substantially higher affinity than conventional soluble VEGF receptors. VEGF Trap bound to all isoforms of human VEGF-A tested with subpicomolar affinity. Ranibizumab and bevacizumab also bound human VEGF-A, but with markedly lower affinity. The association rate for VEGF Trap binding to VEGF-A was orders of magnitude faster than that measured for bevacizumab and ranibizumab. Similarly, in cell-based bioassays, VEGF Trap inhibited the activation of VEGFR1 and VEGFR2, as well as VEGF-A induced calcium mobilization and migration in human endothelial cells more potently than ranibizumab or bevacizumab. Only VEGF Trap bound human PlGF and VEGF-B, and inhibited VEGFR1 activation and HUVEC migration induced by PlGF. These data differentiate VEGF Trap from ranibizumab and bevacizumab in terms of its markedly higher affinity for VEGF-A, as well as its ability to bind VEGF-B and PlGF.

**Electronic supplementary material:**

The online version of this article (doi:10.1007/s10456-011-9249-6) contains supplementary material, which is available to authorized users.

## Introduction

Angiogenesis is the process by which new vessels are created from pre-existing vasculature. Abnormal angiogenesis is a hallmark of diseases such as cancer [1] and the neovascular or ‘wet’ form of age-related macular degeneration (AMD) [2], the leading cause of blindness in the elderly population [3]. The process is characterized by an increase in the number of proliferating endothelial and stromal cells, and altered morphology of the vasculature [4, 5]. Several proangiogenic factors are consistently upregulated during diverse forms of pathological angiogenesis, including two members of the vascular endothelial growth factor (VEGF) family, VEGF-A and placental growth factor (PlGF) [6–8]. These factors activate quiescent endothelial cells and promote cell proliferation, migration and vascular permeability [5–9]. As in cancer, VEGF-A is the major driver of pathological angiogenesis and vascular leak in wet AMD, as well as in other ocular vascular diseases, such as diabetic and ischemic retinopathies. Moreover, growing evidence suggests that PlGF synergizes with VEGF-A in promoting vascular pathology in these diverse conditions [10–16].

In humans and other mammals, the VEGF family of factors consists of five related glycoproteins, VEGF-A, -B, -C, -D and PlGF [17, 18]. VEGF-A is the first, and most well studied member of the VEGF family and is currently a key target for antiangiogenic therapy [17]. Although encoded by a single gene, several distinct isoforms of VEGF-A exist as a result of alternative splicing and/or proteolytic cleavage. The various VEGF-A isoforms are all active as dimers, differing principally in their size and their ability to bind heparin or accessory, non-signaling binding proteins called neuropilins. For example, VEGF-A_165_ binds heparin and neuropilins with low affinity, and is the predominant isoform expressed in humans. VEGF-A_121_ is also expressed at high levels in many tissues and in pathological conditions, but it lacks the domains that mediate binding to heparin and neuropilins [17, 18] and is thus freely diffusible. Other isoforms such as VEGF-A_189_ and VEGF-A_206_ bind heparin with high affinity and thus accumulate in the extracellular matrix. Isoforms of VEGF-B and PlGF, which differ in their capacity to bind heparin and/or neuropilins are also produced by alternative splicing.

VEGF family ligands bind with high affinity to and signal through three receptor tyrosine kinases, VEGFR1, VEGFR2 and VEGFR3 [8, 17–19]. VEGFR2 is expressed predominantly on vascular endothelial cells. In addition to being expressed on the vascular endothelium, VEGFR1 is also expressed by several other cell types including neutrophils, monocytes, macrophages, mural cells, and endothelial progenitor cells. Although VEGFR1 has a higher affinity for VEGF-A than does VEGFR2, in endothelial cells VEGFR1 exhibits only weak tyrosine phosphorylation when activated by VEGF-A induced dimerization. Thus, the effects of all isoforms of VEGF-A on the vascular endothelium are thought to be mediated primarily through activation of VEGFR2. PlGF and VEGF-B bind only to VEGFR1, and in further contrast to VEGF-A, neither PlGF nor VEGF-B are essential for normal vascular development or physiological angiogenesis in the adult. However, like VEGF-A, both PlGF and VEGF-B have been implicated in pathological vascular remodeling [8, 11, 18]. The remaining VEGF family members, VEGF-C and VEGF-D, bind with high affinity to VEGFR3. VEGFR3 is found primarily on lymphatic endothelial cells in the adult. Consequently, VEGF-C and VEGF-D are involved primarily in the regulation of lymphangiogenesis [19], although VEGFR3 signaling is also thought to be important for both developmental and tumor angiogenesis [20–22].

The arsenal of VEGF blockers has evolved over time, with newer generations offering potentially improved anti-angiogenic activity by increasing their affinity for VEGF-A, and/or the number of VEGF-isoforms and family members that they inhibit. Pegaptanib (Macugen™, Eyetech, Inc.) is an aptamer that selectively binds to and neutralizes VEGF-A_165_, but not VEGF-A_121_, and was the first anti-VEGF therapy approved for the treatment of wet AMD [23, 24]. Bevacizumab (Avastin^®^, Genentech, Inc.) is a recombinant, humanized monoclonal antibody that binds all isoforms of VEGF-A, and has been approved for the treatment of metastatic colorectal cancer, non-small-cell lung cancer, and glioblastoma multiforme [1, 25]. Ranibizumab (Lucentis^®^, Genentech, Inc.) was developed specifically for intravitreal administration to treat vascular eye diseases, notably the wet or neovascular form of AMD [26, 27]. Ranibizumab is an affinity-matured antigen-binding fragment (Fab) derived from bevacizumab, and thus has a higher affinity for VEGF-A relative to that of the parental bevacizumab Fab molecule (Fab-12) [28]. Ranibizumab was developed as a Fab because the smaller size was thought to enhance its diffusion from the vitreous into the retina and choroid, relative to full-length antibodies [26]. Being an antibody Fab fragment, each ranibizumab molecule has one binding site for VEGF (compared to bevacizumab’s two), such that two molecules of ranibizumab are bound by each VEGF dimer. In clinical trials, pegaptanib was shown to have a modest effect in slowing the rate of vision loss in patients with wet AMD, while ranibizumab has proven to be highly effective not only in reducing macular edema and preventing further vision loss, but also in producing clinically meaningful improvements in vision in significant numbers of patients [26, 29, 30]. Ranibizumab has been approved by the FDA for the treatment of wet AMD, while bevacizumab is also currently used off-label to treat AMD by intravitreal administration. While the comparative safety and efficacy of bevacizumab for the treatment of wet AMD have not yet been definitively established, several large, controlled clinical trials comparing the relative efficacy of ranibizumab and bevacizumab in the wet AMD are in progress [31, 32].

VEGF Trap (aflibercept, Regeneron Pharmaceuticals, Inc.) is a novel type of soluble decoy receptor generated with Trap technology [33], which employs the fusion of components from multiple endogenous receptors. VEGF Trap consists of an all human amino-acid sequence and comprises the second Ig domain of human VEGFR1 and the third Ig domain of human VEGFR2 expressed as an inline fusion with the constant region (Fc) of human IgG1 [34]. Like bevacizumab and ranibizumab, VEGF Trap binds multiple isoforms of VEGF-A [35] but in contrast to these antibodies the VEGF Trap was designed to also bind the related VEGFR1 ligands, VEGF-B and PlGF. An intravenous formulation of VEGF Trap, generically known as aflibercept, is being developed for use in oncology [ZALTRAP™ (aflibercept)]; this formulation is hyperosmotic and diluted prior to infusion. An alternate formulation of aflibercept, known as VEGF Trap-Eye [EYLEA™ (aflibercept) Injection)], is an ultra-purified and iso-osmotic drug product that has been developed specifically for intravitreal injection for use in the treatment of various ophthalmological conditions.

Although some data on the binding affinities and in vitro activities of bevacizumab, ranibizumab and VEGF Trap have been published [28, 34, 36–40], the available data are incomplete. Moreover, comparison of the currently available data for these agents across publications is problematic as the experimental methods, cell lines, and particular conditions employed differ significantly from study to study. For example, the equilibrium dissociation constant (*K*_D_) of the Fab fragment of bevacizumab (Fab-12) for VEGF-A has been variously reported as 1.8 and 20 nM, as determined by surface plasmon resonance (SPR) technology (Biacore) [28, 36], while the binding characteristics of the full bivalent bevacizumab molecule have not been reported. Thus, the goal of the present work was to assess the binding properties and in vitro activity of VEGF Trap, ranibizumab and bevacizumab under identical experimental conditions.

The results of these experiments show that VEGF Trap binds to VEGF-A with higher affinity and a faster association rate than ranibizumab or bevacizumab, and that VEGF Trap has the unique ability to additionally bind VEGF-B and PlGF. Consistent with its higher affinity for VEGF-A and faster association rate, VEGF Trap demonstrates increased potency relative to ranibizumab and bevacizumab in blocking VEGF-A induced activation of VEGFR1 and VEGFR2 in cell-based assays, and also in blocking VEGF-mediated calcium mobilization and migration in human endothelial cells. Finally, the high affinity binding of VEGF Trap to PlGF is borne out by the finding that only VEGF Trap can markedly inhibit VEGFR1 activation and endothelial cell migration induced by PlGF.

## Materials and methods

### VEGF reagents

Human VEGF-A_121_, human PlGF-1, human VEGF-C, human VEGF-D, murine VEGF-A_164_, murine VEGF-A_120_, murine PlGF-2, rat VEGF-A_164_, human VEGFR1-hFc, human VEGFR2-hFc and hVEGFR3-hFc were purchased from R&D Systems (Minneapolis, MN). VEGF Trap, rabbit VEGF-A_165_, human PlGF-2, human VEGF-B_(10-108)_ and human VEGF-A_165_ were made at Regeneron Pharmaceuticals, Inc. (Tarrytown, NY). Bevacizumab and ranibizumab (Genentech, Inc., South San Francisco, CA) were purchased.

### Surface plasmon resonance (SPR)

SPR experiments were performed on a Biacore 3000 instrument using a dextran-coated (CM5) chip at 25°C. The running buffer was filtered HBS-T (10 mM Hepes, 150 mM NaCl, 3.4 mM EDTA, 0.05% polysorbate 20, pH 7.4). A capture sensor surface was prepared by covalently immobilizing recombinant Protein A (Pierce, Rockford, IL) or an anti-human Fab polyclonal antibody (human Fab capture kit, GE Healthcare, Piscataway, NJ) to the chip surface using (1-Ethyl-3-[3-dimethylaminopropyl]carbodiimide hydrochloride)/N-hydroxysuccinimide (EDC/NHS) coupling chemistry. Following surface activation, Protein A or anti-human Fab polyclonal antibody in coupling buffer (0.1 M acetate buffer, pH 4.5) was injected over the activated chip surface until a resonance unit (RU) signal of about 2,000 RU (Protein A) or 1,000 RU (anti-human Fab polyclonal antibody) was reached. The activated coupled chip surfaces were then washed and treated with 10 mM glycine–HCl, pH 1.5, to remove uncoupled residual proteins.

VEGF Trap, bevacizumab or ranibizumab were diluted into the running buffer and captured on the coupled Protein A (VEGF Trap and bevacizumab) or anti-human Fab polyclonal antibody (ranibizumab) chip surface. Following the capture step, a range of concentrations of test ligands (1.0–0.062 nM for VEGF-A ligands, 2.5–0.156 nM for VEGF-B_(10-108)_ and 5.0–0.078 nM for PlGF ligands) were individually injected over VEGF inhibitor captured surfaces. For all ligands, the association rate constant (*k*_a_) was determined from data obtained at multiple test ligand concentrations. The dissociation rate constant (*k*_d_), which is independent of test ligand concentration, was determined from the change in VEGF inhibitor-bound test ligand RU over time (~10–70 min) for PlGF and VEGF-B ligands. Since the dissociation rate (*k*_d_) of VEGF-A family ligands is too slow to allow for sufficient RU change within ligand dissociation time periods typically employed, the dissociation rates for these ligands were measured on a Biacore 2000 instrument using the “fixed *k*_d_” procedure as described by Drake et al. [41]. This format uses a saturating concentration of ligand for binding, followed by monitoring the dissociation rate for an extended period of time (~2–3 h). Specific Biacore kinetic sensorgrams (Online Resource 1, Figures 1–5) were obtained by a double referencing procedure as described by Myszka et al. [42]. The data were then processed using Scrubber software (version 2.0, BioLogic Software) and kinetic analyses performed using BiaEvaluation (version 4.1, Biacore). The equilibrium dissociation constant (*K*_D_) was calculated from the ratio of the dissociation rate constant divided by the association rate constant (*K*_D_ = *k*_d_/*k*_a_). Similar studies were conducted to evaluate the binding kinetics of VEGF-A_165_ to the extracellular domains of native VEGFR1 and VEGFR2 fused to human Fc (Online Resource, Fig. 5) and several other VEGF family related ligands from multiple species (Online Resource 1, Table 1). Additional studies demonstrated no detectable binding of VEGF Trap to human VEGF-C and human VEGF-D, however a positive control binding experiment confirmed the ability of VEGF-C and VEGF-D to associate with VEGFR3 (Online Resource 1, Fig. 5).

### KinExA equilibrium assays

In addition to surface capture kinetic experiments, solution binding studies were also conducted at room temperature (25°C) using a KinExA 3000 instrument (Sapidyne Instruments, Boise, ID) to quantify the equilibrium binding constants of VEGF inhibitors in solution, using varying concentrations of VEGF-A_165_,VEGF-A_121_, hPlGF-2 or VEGF-B_(10-108)_. Inhibitor-ligand mixtures were equilibrated at room temperature for 10–96 h. Fifty microgram of human VEGF-A_165_ was immobilized onto 75 mg Azlactone beads, suspended in 1.5 ml PBS and rotated at 4°C overnight. The supernatant was removed and the beads were incubated for another hour at room temperature in 1.0 ml PBS with 10 mg/ml BSA to block nonspecific binding sites. The blocked beads were washed three times with PBS, resuspended in 30 ml of PBS, and used immediately. Co-complex mixtures contained: VEGF Trap (concentration range 1–50 pM) with VEGF-A_165_ or VEGF-A_121_ (concentration range 19.5 fM–100 pM) or hPLGF-2 (concentration range 0.5pM–5 nM) or VEGF–B_(10–108)_ (concentration range 0.61 pM–1.25 nM): Ranibizumab (concentration range 50–400 pM) with VEGF-A_165_ (concentration range 0.73 pM–15 nM): Bevacizumab (concentration range 25–50 pM) with VEGF-A_165_ (concentration range 0.49 pM–5 nM). Human VEGF-A_165_ was coupled to Azlactone beads and was used to capture unbound inhibitor. Equilibrated mixtures were injected through a column of VEGF-A_165_-coupled micro-beads in the KinExA system at a flow rate of 0.25 ml/min. Bead contact time was <0.5 s, permitting unbound VEGF inhibitors to be captured by the beads without perturbing the equilibrium state of the solution. Captured VEGF inhibitors were quantified with Cy5-conjugated goat polyclonal anti-human IgG or anti-human F(ab′)_2_ fragment specific for light-chain antibody (Jackson ImmunoResearch Laboratories, West Grove, PA). The *K*_D_ was obtained from nonlinear regression analysis of the data using a one-site homogeneous binding model contained within the KinExA software (Version 1.0.3; Sapidyne Instruments) using the ‘standard analysis’ method. The software calculates the *K*_D_ and determines the 95% confidence interval by fitting the data points to a theoretical *K*_D_ curve (Online Resource 1, Figures 6 and 7). The 95% confidence interval is given as *K*_D_ low and *K*_D_ high as described by Darling et al. [43].

### Cell-based bioassays

#### VEGFR1/VEGFR2 cell lines and VEGF assay

In order evaluate the ability of VEGF Trap, ranibizumab and bevacizumab to specifically block ligand-mediated dimerization and activation of VEGFR1 or VEGFR2, two separate cell lines expressing these receptors were created. Two chimeric VEGFR1 receptors were constructed that incorporated the VEGFR1 extracellular domain (1–756, Genbank # NP_002010) fused to the transmembrane and cytoplasmic domain of either IL18Rα (328–541, Genbank # NP_003846.1) or IL18Rβ. 355–549, Genbank # NP_003844.1). The VEGFR1/IL18Rα chimeric receptor was cloned into a plasmid with a G418 resistance marker, while the VEGFR1/IL18Rβ chimeric receptor was cloned into a plasmid with a hygromycin resistance marker. The chimeric receptors were transfected into an HEK293 cell line with an integrated NFκB-luciferase-IRES-eGFP reporter gene using Lipofectamine plus (Invitrogen, Carlsbad, CA) according to manufacturer’s instructions. Likewise, similar chimeric receptors incorporating the VEGFR2 extracellular domain (1–764, Genbank # NP_002244.1) fused to the transmembrane and cytoplasmic domain of either IL18Rα or IL18Rβ were constructed and transfected into the same HEK293 reporter cell line. In order to isolate cells for use in a bioassay, the cells were grown in G418 (Invitrogen, Inc.) and hygromycin (Calbiochem) to ensure the presence of both chimeric receptors. Cells underwent further selection by stimulating the cells with VEGF and then sorting cells expressing GFP by fluorescence activated cell sorting (FACS). When the extracellular VEGFR1 or VEGFR2 is dimerized by binding VEGF, the IL18Rα and β intracellular domains interact and are able to signal through the NFκB driven luciferase reporter gene.

#### VEGF and PlGF activation of the VEGFR1 and VEGFR2 cell lines

Cells expressing either VEGFR1 or VEGFR2 were resuspended at 1.25 × 10^5^ cells/ml in Optimem (Invitrogen, Inc.) plus 0.1% fetal calf serum (FCS) and 80 μl was placed in each well of a 96 well plate (10,000 cells/well). The cells were incubated overnight at 37°C, 5% CO_2_. The dose response curve for VEGFR1 activation was determined by adding 20 μl of VEGF-A_165_, VEGF-A_121_ or PlGF-2 (human or mouse) to the cells at concentrations ranging from 0.022 pM to 4.0 nM. One well served as the negative control with no test ligand added. The dose response curve for VEGFR2 activation was determined by adding 20 μl of VEGF-A_165_, VEGF-A_121_, or hPlGF-2 to the cells at the same concentrations used above. Each dose response curve was done in quadruplicate. After addition of the VEGF or PlGF, the plates were incubated at 37°C and 5% CO_2_ for 6 h, and then equilibrated to room temperature for 30 min. An equal volume of One-glo luciferase substrate (Promega, Madison, WI) was added to each well and the plate was incubated at room temperature for a further 15 min. Plates were read on Victor X instrument and the values were analyzed by a four-parameter logistic equation over a 12–point dose response curve (Prism, GraphPad Software, version 5.03, La Jolla, CA).

VEGF Trap, bevacizumab and ranibizumab were tested with both the VEGFR1 and VEGFR2 cell lines. VEGF Trap was added to the cells at concentrations ranging from 0.8 pM to 50 nM and included a control well with buffer. Bevacizumab and ranibizumab were added to the cells at concentrations ranging from 8.5 pM to 500 nM and included a control well. Immediately after addition of VEGF Trap or the antibodies to the VEGFR1 cell line, VEGF-A_165_, VEGF-A_121_, or hPlGF-2 was added to the cells at a constant concentration of 20 pM (VEGF) or 40 pM (hPlGF-2). The VEGFR2 cell line was stimulated with 20 pM VEGF-A_165_ or 20 pM VEGF-A_121_. The plates were incubated at 37°C and 5% CO_2_ for 6 h and then equilibrated to room temperature for 30 min. An equal volume of One-glo luciferase substrate (Promega) was added to each well and the plate was incubated at room temperature for a further 15 min. Plates were read on Victor X instrument and the values were analyzed by a four-parameter logistic equation over a 12-point response curve (GraphPad Prism). Each inhibition curve was done in triplicate.

#### VEGF dependent calcium mobilization in human umbilical vein endothelial cells (HUVEC)

HUVEC (Vec Technologies, Inc., Rensselaer, NY) were diluted to 3 × 10^5^ cells/ml in MCDB-131 complete medium (Vec Technologies, Inc.), and 100 μl was added to each well of a 96 well plate. The plates were incubated overnight at 37°C and 5% CO_2_. The media was then removed and the HUVEC loaded with a calcium sensitive dye, Fluo4 NW (Invitrogen, Inc), in ECB media (BD Biosciences) with 0.25 mM of probenicid and 0.3% BSA (80 μl per well). The solution was incubated with the cells for 30 min at 37°C and 5% CO_2_ followed by another 30 min at room temperature.

To measure the dose response, HUVEC were simulated with buffer or VEGF-A_165_ at concentrations ranging from 0.023 pM to 4.0 nM. The cellular response was recorded at a fluorescence emission wavelength of 575 nm with an excitation of 515 nm for 6 min, using the FLIPR^TETRA^ (Molecular Devices, Sunnyvale, CA). Each dose response curve was done in duplicate.

Inhibition of VEGF-A_165_ was determined by adding VEGF Trap at concentrations ranging from 0.17 pM to 10.0 nM and for bevacizumab and ranibizumab at concentrations ranging from 8.4 pM to 500 nM. VEGF Trap, bevacizumab, or ranibizumab were incubated with 20 pM VEGF-A_165_ for 10 min and then added to the cells, and the calcium response recorded as above. The data were analyzed using the average peak fluorescence at each inhibitor concentration tested in triplicate.

### Cell migration assays

#### Cell culture

HUVEC, at first passage, were purchased from VEC Technologies and grown at 37°C in a 5% CO_2_ humidified incubator, in MCDB-131 complete media. Cells grown to confluency in 10 cm^2^ culture dishes, were washed twice with Hank’s Buffered Saline Solution (HBSS; Mediatech, Manassas, VA.) without calcium, magnesium or phenol red, and dissociated with Trypsin/EDTA (Lonza, Walkersville, MD). Cells were then seeded at approximately 2 × 10^5^ cells/dish and typically reached confluency in 3–4 days. Prior to use in cell migration assays, cells were serum-starved for 5 h in MCDB-131 basal media (MBM; VEC Technologies) supplemented with 2 mM l-glutamine, 100 U/ml Penicillin, 100 μg/ml Streptomycin, 10 μg/ml heparin, and 0.1% fetal bovine serum.

#### HUVEC migration

HUVEC migration was assessed using a modified Boyden chamber [BD FluoroBlok™ 24-well Biocoat angiogenesis system: Endothelial cell migration (ECM); 3 μm pore size] according to the manufacturer’s suggested protocol. Briefly, serum-starved HUVECs were dissociated using enzyme-free cell dissociation media (Millipore, Billerica, MA) and resuspended in MBM to a final concentration of 2–3 × 10^5^ cells/ml. An aliquot of resuspended cells (250 μl; ~50,000 cells/well) was placed in the upper well of the ECM plate, and MBM (750 μl) with or without ligand (130 pM human VEGF-A_165_, 7.1 nM human PLGF-2, or 3.5 nM mouse PLGF-2), was mixed with VEGF Trap, bevacizumab, or ranibizumab (inhibitor concentration range 0.013–13 nM) and placed in the lower well following a 1 h incubation of the mixture at room temperature. The ECM plate was incubated for 18–20 h in a 37°C/5% CO_2_ incubator to allow cells from the upper well to migrate through the FluoroBlok™ membrane towards the lower well. Following migration, cells attached to the underside of the FluoroBlok™ membrane were stained with 500 μL of a 2 μg/mL solution of the fluorescent dye Calcein AM (Anaspec, Freemont, CA) for 1.5 h in a 37°C/5% CO_2_ incubator. Fluorescence emission was measured at 580 nm with excitation at 485 nm in a Flexstation 3 (Molecular Devices, Sunnyvale CA) bottom-reading fluorescent plate reader. Statistical analyses were carried out using a 1-way ANOVA followed by a Dunnett’s multiple comparison post hoc test (Prism, GraphPad Software, version 5.03, La Jolla, CA).

## Results

### VEGF Trap binds VEGF-A, VEGF-B and PlGF from multiple species with high affinity

The interaction between VEGF Trap and VEGF family ligands was measured using SPR-Biacore technology. Kinetic binding data was generated using an amine-coupled Protein A surface and subsequent VEGF Trap capture at low density. VEGF Trap bound heparin binding and non-heparin binding isoforms of human VEGF-A, and PlGF, as well as VEGF-B_(10-108)_ with high affinity (Table 1 and Online Resource 1, Table 1). Notably, the equilibrium dissociation constant (*K*_D_) of VEGF Trap for VEGF-A_165_ (0.490 pM) was significantly lower (tighter binding) than that of the extracellular domains of dimerized human VEGFR1 (9.33 pM) or VEGFR2 (88.8 pM) fused inline to hFc (Table 1 and Online Resource 1, Fig. 5). The above absolute and relative *K*_D_ values for VEGFR1-Fc and VEGFR2-Fc are comparable to those previously reported for native VEGFR1 and VEGFR2 using cell-based bindings assays [44, 45]. VEGF Trap did not bind human VEGF-C or human VEGF-D (Online Resource 1, Fig. 5). The *K*_D_ values for the interaction between VEGF Trap and VEGF-A from mouse, rat and rabbit were similar to those of human and ranged from 0.471 to 0.776 pM. VEGF Trap also bound human and murine PlGF-2 with a *K*_D_ of 38.9 and 3.32 pM, respectively (Table 1 and Online Resource 1, Table 1). In contrast, bevacizumab and ranibizumab are specific for human and non-human primate VEGF-A, and do not effectively bind or neutralize rodent VEGF [46–48].

**Table 1:** Kinetic binding parameters for VEGF Trap, ranibizumab and bevacizumab binding to human VEGF family ligands determined by SPR-Biacore

VEGF inhibitor	Ligand	Kinetic binding parameters		
		*k*_a_/10^5^ (M^−1 ^s^−1^)	*k*_d_/10^−5^ (s^−1^)	*K*_D_ (pM)
VEGF Trap^a^	VEGF-A_121_	375.0 (5.0)	1.35 (.02)	0.360
VEGF Trap^a^	VEGF-A_165_	410.0 (10.0)	2.01 (.01)	0.490
Ranibizumab^b^	VEGF-A_165_	1.6 (0.003)	0.73 (.005)	46
Bevacizumab^a^	VEGF-A_165_	5.3 (0.01)	3.10 (.02)	58
hVEGFR1-Fc^a^	VEGF-A_165_	300.0 (20.0)	28.0 (1.0)	9.33
hVEGFR2-Fc^a^	VEGF-A_165_	152.0 (5.0)	135 (6.0)	88.8
VEGF Trap^a^	PlGF-2	17.5 (0.06)	6.81 (.03)	38.9
Ranibizumab^b^	PlGF-2	NB	NB	NB
Bevacizumab^a^	PlGF-2	NB	NB	NB
VEGF Trap^a^	VEGF-B_(10-108)_	352.0 (3.0)	6.74 (.09)	1.92

Numbers in parentheses represent the standard error of the kinetic fit

*NB* No binding under assay conditions used

^a^VEGF inhibitor captured on a Protein A-coupled sensor chip

^b^VEGF inhibitor captured on an anti-human Fab polyclonal antibody-captured sensor chip

### Binding parameters for VEGF Trap, ranibizumab and bevacizumab interactions with human VEGF-A_165_ and PlGF-2

While all three VEGF inhibitors bound human VEGF-A_165_ with high affinity, the *K*_D_ for VEGF Trap binding of VEGF-A_165_ was approximately 100-fold lower (i.e. the binding affinity was ~100-fold tighter) than that for ranibizumab or bevacizumab (Table 1). Specifically, the *K*_D_ value for VEGF Trap was 0.490 pM, while those for ranibizumab and bevacizumab were 46 and 58 pM, respectively. The lower *K*_D_ value for VEGF Trap binding VEGF-A_165_ was primarily attributable to a significantly faster association rate (*k*_a_) that was 77- and 256-fold faster than that for bevacizumab and ranibizumab, respectively (Table 1). VEGF Trap also bound human PlGF-2 with high affinity (*K*_D_ = 38.9 pM), whereas no binding was detected between ranibizumab or bevacizumab and human PlGF-2 (Table 1). Biacore kinetic sensorgrams analyzed for association and dissociation rate constants are provided in Online Resource 1, Figures 1–4.

To confirm the surface kinetic data determined using SPR-Biacore, the binding interactions between soluble VEGF Trap, bevacizumab or ranibizumab and human VEGF-A_165_ were also compared in solution equilibrium assays using KinExA methodology. As shown in Table 2 and Online Resource 1, Figures 6 and 7, the absolute *K*_D_ values and 95% confidence interval obtained for the VEGF inhibitors binding to VEGF-A_165_, were comparable to those obtained with SPR-based measurements. Similarly, VEGF Trap binding affinities for VEGF-A_121_, VEGF-B_(10-108)_, and PlGF were also comparable between SPR and solution based equilibrium assays.

**Table 2:** Solution binding parameters for VEGF Trap, ranibizumab and bevacizumab binding to human VEGF family ligands determined by KinExA equilibrium assays

VEGF inhibitor	Ligand	Kinexa equilibrium binding parameters	
		*K*_D_ (pM)	*K*_D_ range (pM)^a^
VEGF Trap	VEGF-A_165_	0.66	0.36–1.06
Ranibizumab	VEGF-A_165_	20.6	10.9–36.3
Bevacizumab	VEGF-A_165_	35.1	12.2–82.9
VEGF Trap	VEGF-A_121_	0.18	0.08–0.32
VEGF Trap	PlGF-2	20.7	13.7–29.3
VEGF Trap	VEGF-B_(10-108)_	17.5	12.9–22.9

^a^95% confidence interval

### Effects of VEGF Trap, ranibizumab and bevacizumab on VEGF-A or PlGF-2 induced activation of VEGFR1

**Fig. 1:**
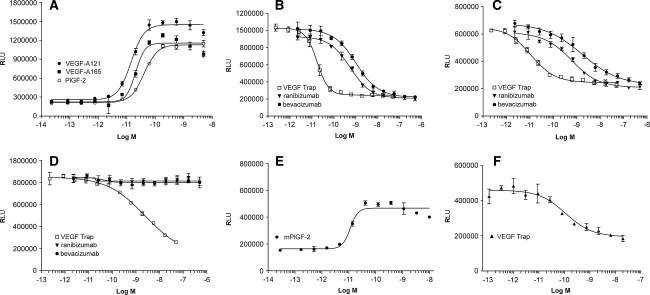
The effects of VEGF Trap, ranibizumab and bevacizumab on luciferase activation induced by VEGF-A_121_, VEGF-A_165_, human PlGF-2 (hPlGF-2) or mouse PlGF-2 (mPLGF-2) in HEK293/VEGFR1 cells. **a** Dose response curves for VEGF-A_121_, VEGF-A_165_ and hPlGF-2 yielded EC_50_ values of 13, 17, and 29 pM, respectively. **b** Serial dilutions of VEGF Trap (*open box*), ranibizumab (*triangle*), or bevacizumab (*closed circle*) were added to HEK293/VEGFR1 cells along with 20 pM of VEGF-A_121_. **c** Serial dilutions of VEGF Trap (*open box*), ranibizumab (*triangle*), or bevacizumab (*closed circle*) were added to HEK293/VEGFR1 cells along with 20 pM of VEGF-A_165_. **d** Serial dilutions of VEGF Trap (*open box*), ranibizumab (*triangle*), or bevacizumab (*closed circle*) were added to HEK293/VEGFR1 cells along with 40 pM of human PlGF-2. **e** Dose response curve for mPlGF-2 yielded an EC_50_ value of 10 pM (**f**). Serial dilutions of VEGF Trap were added to HEK293/VEGFR1 cells along with 20 pM of mPlGF-2. The cells were incubated for 6 h and OneGlo luciferase substrate was then added to each well. The plates were read on a luminometer and the data were plotted using a four parameter curve fit with GraphPad Prism. Each point represents a replica of 3 wells at each concentration

To determine the ability of VEGF Trap, ranibizumab and bevacizumab to block human VEGF-A or PlGF-2 induced VEGFR1 activation in vitro, a VEGFR1 specific luciferase assay was developed, which used the human cell line HEK293 transfected with an NFκB-luciferase reporter plasmid and human VEGFR1 (Fig. 1). Notably in this assay, the potency of ranibizumab for blocking 20 pM VEGF-A_121_ or VEGF-A_165_ induced luciferase activity through VEGFR1 was only slightly greater than that of bevacizumab. Ranibizumab exhibited IC_50_ values (50% inhibitory concentration) of 675 and 1,140 pM, while IC_50_ values for bevacizumab were 845 and 1,476 pM for VEGF-A_121_ or VEGF-A_165_, respectively. In contrast, VEGF Trap exhibited a 45–92-fold greater blocking potency compared to either ranibizumab or bevacizumab, with IC_50_ values of 15 and 16 pM for blocking VEGFR1 activation by 20 pM VEGF-A_121_ or VEGF-A_165_, respectively (Table 3; Fig. 1). VEGF Trap also blocked luciferase activity induced by human PLGF-2 (40 pM) or mouse PlGF-2 (20 pM) with IC_50_ values of 2.9 nM and 104 pM, respectively. In contrast, neither bevacizumab nor ranibizumab showed ability to block human or mouse PlGF-2 under these experimental conditions.

**Table 3:** Summary of IC_50_ values for VEGF Trap, ranibizumab and bevacizumab blocking VEGF-A or PlGF-2 induced activation of VEGFR1 and VEGFR2

VEGF inhibitor	VEGFR1 cell line				VEGFR2 cell line		Ca^2+^ mobilization in HUVE cells
	IC_50_ at 20 pM hVEGF-A_121_	IC_50_ at 20 pM hVEGF-A_165_	IC_50_ at 40 pM hPlGF-2	IC_50_ at 20 pM mPlGF-2	IC_50_ at 20 pM hVEGF-A_121_	IC_50_ at 20 pM hVEGF-A_165_	IC_50_ at 20 pM hVEGF-A_165_
VEGF Trap	15 pM (2.4)	16 pM (2.2)	2,890 pM (227)	104 pM (23)	16 pM (2.5)	26 pM (11)	2.6 pM (1.2)
Ranibizumab	675 pM (165)	1,140 pM (226)	NB	NB	576 pM (84)	845 pM (185)	334.9 pM (61.1)
Bevacizumab	854 pM (214)	1,476 pM (288)	NB	NB	630 pM (66)	1,323 pM (491)	70.8 pM (20.1)

Numbers in parentheses represent standard error of the mean

The IC_50_ numbers were obtained from at least 3 separate experiments

hVEGF: human VEGF; hPlGF-2: human PlGF-2; mPLGF-2: mouse PlGF-2

*NB* No blocking activity observed under the assay conditions used

### Effects of VEGF Trap, ranibizumab and bevacizumab on VEGF-A induced activation of VEGFR2

**Fig. 2:**
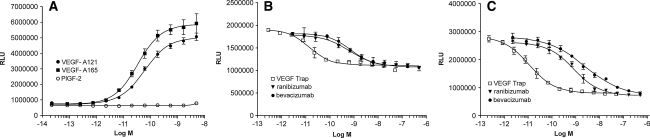
The effects of VEGF Trap, ranibizumab and bevacizumab on luciferase activation induced by VEGF-A_121_ and VEGF-A_165_ in HEK293/VEGFR2 cells. **a** Dose response curves for VEGF-A_121_ and VEGF-A_165_ with EC_50_ values of 70 and 30 pM, respectively. PlGF-2 was not active in this assay. **b** Serial dilutions of VEGF Trap (*open box*), ranibizumab (*triangle*) or bevacizumab (*closed circle*) were added to HEK293/VEGFR2 cells along with 20 pM of VEGF-A_121_. **c** Serial dilutions of VEGF Trap (*open box*), ranibizumab (*triangle*) or bevacizumab (*closed circle*) were added to HEK293/VEGFR2 cells along with 20 pM of VEGF-A_165_. The cells were incubated for 6 h and OneGlo luciferase substrate was then added to each well. The plates were read on a luminometer and the data were plotted using a four parameter curve fit with GraphPad Prism. Each point represents a replica of 3 wells at each concentration

To determine the ability of VEGF Trap, ranibizumab and bevacizumab to block VEGFR2 activation in vitro, a VEGFR2 specific luciferase assay was developed, which used the human cell line HEK293 transfected with an NFκB-luciferase reporter plasmid and human VEGFR2 (Fig. 2). As for VEGFR1, VEGF Trap efficiently blocked VEGFR2 signaling induced by 20 pM of human VEGF-A_121_ or VEGF-A_165_ (IC_50_ of 16 and 26 pM, respectively). VEGF Trap was again markedly more potent in blocking VEGF-mediated VEGFR2 activation than either ranibizumab or bevacizumab (33–51-fold more potent, see Fig. 2; Table 3). As expected, hPlGF-2 was not able to activate VEGFR2 in this assay.

### The effect of VEGF Trap, ranibizumab and bevacizumab on VEGF-A_165_ induced calcium mobilization in human endothelial cells

**Fig. 3:**
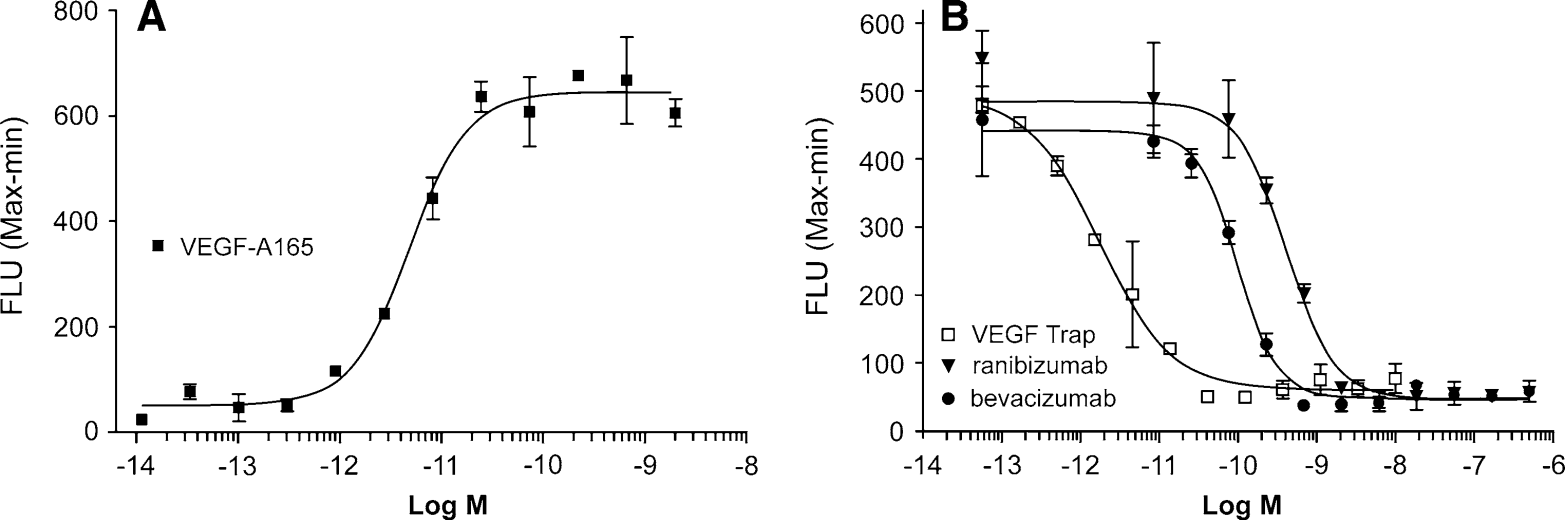
The effects of VEGF Trap, ranibizumab and bevacizumab on calcium mobilization induced byVEGF-A_165_ in HUVEC. **a** A dose–response curve generated using serial dilutions of VEGF-A_165_ (4.0 nM–0.023 pM) resulted in an EC_50_ value of 5 pM. **b** Serial dilutions of VEGF Trap (*open box*), ranibizumab (*triangle*) or bevacizumab (*closed circle*) were added to HUVEC along with 20 pM of VEGF-A_165_. The VEGF-A_165_ was preincubated with the inhibitors for 10 min at 25°C. The solution was added to HUVEC preloaded with fluo-4 and the fluorescence of the well was determined on a FLIPR instrument. The data were plotted using a four parameter curve fit with GraphPad Prism. Each point represents duplicate wells at each concentration

The ability of the three VEGF inhibitors to block human VEGF-A_165_ induced activation of VEGF receptors was also tested in human endothelial cells. A VEGF-A_165_ induced calcium mobilization assay was developed using HUVEC [49, 50], which express native VEGFR1 and VEGFR2 (Fig. 3). Interestingly in this assay, bevacizumab was ~5-fold more potent than ranibizumab at blocking VEGF-A_165_ induced calcium mobilization. Nevertheless, the IC_50_ for VEGF Trap was ~27-fold lower than that of bevacizumab and ~129-fold lower than ranibizumab, confirming the greater potency of VEGF Trap for blocking VEGFR1 and VEGFR2 activation in vitro (Table 3; Fig. 3). The relative potency of VEGF blockers in this acute assay may reflect differences in their association rate constants.

### The effect of VEGF Trap, bevacizumab and ranibizumab on HUVEC migration induced by VEGF_165_ or PlGF-2

**Fig. 4:**
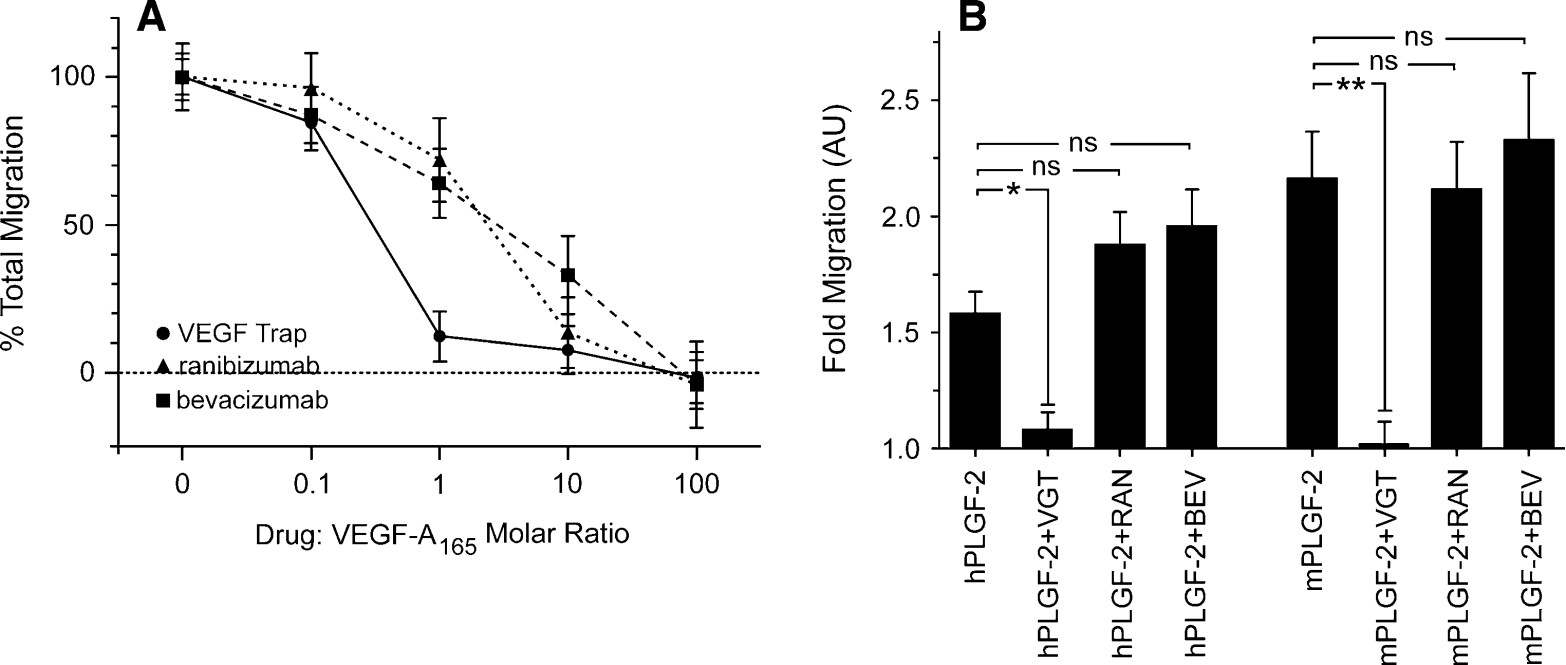
The effects of VEGF Trap, ranibizumab and bevacizumab on HUVEC migration. **a** HUVEC were placed in the upper compartment of the Boyden chamber and allowed to migrate towards basal media containing 0.1% fetal bovine serum with or without VEGF-A_165_ or VEGF-A_165_ mixed with four concentrations each of VEGF Trap (*circles*, *solid line*), ranibizumab (*triangles*, *dotted line*) or bevacizumab (*squares*, *dashed line*) ranging from 0.013 to 13 nM. The percentage of total migration (*y*-axis) was calculated as (*F*_Drug _− *F*_Basal_)/(*F*_Total_− *F*_Basal_) × 100; where *F*_Total_ is fluorescence in the presence of VEGF-A_165_, *F*_Basal_ is fluorescence in the absence of VEGF-A_165_, and *F*_Drug_ is fluorescence in the presence of VEGF-A_165_ mixed with drug at a specific molar ratio (*x*-axis). **b** HUVEC migration was assessed in the absence and presence of human PLGF-2 (hPLGF-2) or mouse PLGF-2 (mPLGF-2) with and without a 100-fold molar excess of VEGF Trap (VGT), ranibizumab (RAN) or bevacizumab (BEV). Fold migration (*y*-axis) was calculated as the ratio *F*/*F*_Basal_; where *F* is the total fluorescence measured for the indicated condition (*x*-axis) and *F*_Basal_ is the fluorescence in the absence of either hPLGF-2 or mPLGF-2. Statistical significance: *P < 0.05; **P < 0.01; ns, no significance. Values and *error bars* represent the average value and standard error of the mean from at least three independent experiments with each experiment containing four biological replicates per condition (total *n* = 12–16 per condition) for all conditions tested. *AU* arbitrary units

Endothelial cell migration plays a central part in the process of angiogenesis and, consistent with its pro-angiogenic profile, VEGF acts as a chemoattractant for endothelial cells [51]. To determine the ability of VEGF Trap, ranibizumab and bevacizumab to block human VEGF-A_165_ induced cell migration, HUVEC mobility was assessed in a modified Boyden chamber assay. None of the VEGF inhibitors affected basal endothelial cell migration in the absence of test ligands (data not shown). In the presence of VEGF-A_165_ (130 pM), VEGF Trap blocked VEGF-A_165_ induced cell migration in a dose-dependent manner (Fig. 4). At a 1:1 molar ratio of VEGF Trap and VEGF-A_165_, cell migration was reduced by approximately 90%. Ranibizumab and bevacizumab also inhibited cell migration in a dose–dependent manner (Fig. 4) but were less potent than VEGF Trap, requiring a 10- to 100-fold greater molar concentration of inhibitor to produce an equivalent level of inhibition of cell migration due to VEGF-A_165_ activation.

PlGF also acts as a chemoattractant for endothelial cells through VEGFR1 [52]. Again, the modified Boyden chamber assay was used to test the ability of the VEGF inhibitors to block HUVEC migration stimulated by human PlGF-2. As shown in Fig. 4 (inset), a 100-fold excess of VEGF Trap blocked cell migration induced by human PlGF-2 (7.1 nM) or mouse PlGF-2 (3.5 nM) by approximately 80%. In contrast, ranibizumab and bevacizumab did not inhibit cell migration induced by either human or mouse PlGF–2.

## Discussion

The experiments described herein provide a comprehensive assessment of the ability of VEGF Trap, ranibizumab and bevacizumab to bind and block the activity of VEGF family ligands in vitro, under identical experimental conditions. The data demonstrate that VEGF Trap binds human VEGF-A with higher affinity and a significantly faster association rate, thus neutralizing VEGF-A with greater potency than ranibizumab or bevacizumab. In addition, the studies show that VEGF Trap has the unique ability to bind the additional VEGF family ligands, VEGF-B and PlGF. Moreover, VEGF Trap also bound VEGF-A and PlGF isoforms from all mammalian species tested with similar high affinity, while neither ranibizumab nor bevacizumab efficiently bind and neutralize mouse or rat VEGF-A [46–48].

Several published papers have provided binding affinity data for ranibizumab’s interactions with human VEGF-A [28, 36, 37]. However, to date, binding affinity and specificity data have been provided only for the monovalent Fab fragment of bevacizumab (Fab-12), and not the full bivalent bevacizumab molecule itself. The equilibrium dissociation constant (*K*_D_) for Fab-12 has been variously reported as 1.8 nM [36] or 20 nM [28], indicating an affinity improvement of ranibizumab over Fab-12 of 10–100-fold. Likewise, ranibizumab has been reported to be 30–100-fold more potent than Fab-12 in bioassays measuring VEGF-induced endothelial cell mitogenesis [26]. However, measuring the kinetic binding parameters or in vitro activity of the Fab-12 fragment does not take into account potential avidity interactions of bivalent antibodies, especially when the binding partner is a dimeric ligand such as VEGF-A. These types of avidity driven interactions can significantly increase binding affinity, and potentially the potency of the bivalent antibody relative to that of the monovalent antigen binding fragment in cell-based assays and in vivo.

In the present study, Biacore and KinExA analyses have demonstrated that the equilibrium dissociation constants for VEGF Trap binding VEGF-A_121_ and VEGF-A_165_, were less than 1 pM, in close agreement with earlier reports [34]. In contrast, ranibizumab exhibited a *K*_D_ of 46 pM for VEGF-A_165_. While this represents an approximately 3–4-fold greater affinity for VEGF-A relative to SPR Biacore values previously reported for ranibizumab (*K*_D_ ≤ 140 pM, [28]; ≤179 pM, [37]), it is nevertheless an ~94-fold weaker binding for VEGF-A_165_ relative to VEGF Trap (0.490 pM) (Table 4). Similarly, the *K*_D_ of soluble VEGF Trap for VEGF-A_165_, as determined by KinExA was 0.66 pM, while that of ranibizumab was 20.6 pM, approximately 30-fold lower than that of VEGF Trap.

**Table 4:** Relative VEGF binding affinities and potency of VEGFR signaling blockade

Parameter	Ranibizumab	Bevacizumab	VEGF Trap
*Affinity for VEGF-A*_*165*_ *(Biacore)*	1.0	0.79	94.0
Potency of blocking VEGF (20 pM) mediated signaling			
VEGFR1			
VEGF-A_121_	1.0	0.79	45.0
VEGF-A_165_	1.0	0.77	71.3
VEGFR2			
VEGF-A_121_	1.0	0.91	36.0
VEGF-A_165_	1.0	0.64	32.5
HUVEC			
VEGF-A_165_	1.0	4.73	128.8

The relative fold differences for the *K*_D_ and IC_50_ values for bevacizumab and VEGF Trap are expressed relative to values for ranibizumab (set at 1). Higher numbers reflect tighter binding or increased potency in the indicated assays. Raw values used to calculate relative fold differences were taken from Table 1 and Table 3

Interestingly, the *K*_D_ of bevacizumab for VEGF-A_165_ as determined by Biacore was 58 pM, markedly lower than that reported previously for Fab-12 [28, 36] and within twofold of the binding affinity of ranibizumab. This was also the case for soluble equilibrium binding of bevacizumab in the Kinexa assay (*K*_D_ of 35.1 pM for bevacizumab and 20.6 pM for ranibizumab), and most likely reflects avidity interactions of the bivalent, full antibody molecule. However, like other conventional antibodies that bind dimeric targets, bevacizumab has the potential to form higher order complexes with VEGF, which under some conditions may act as immune complexes [53]. In contrast, each molecule of VEGF Trap forms an inert 1 to 1 complex with VEGF, and cannot form higher order complexes [35].

The *K*_D_ for VEGF Trap binding of VEGF-A documented in the SPR Biacore and KinExA assays translated into increased potency relative to ranibizumab and bevacizumab in all of the bioassays employed. Specifically, VEGF Trap was ~33–71-fold more potent than ranibizumab at inhibiting VEGF-A induced receptor activation in cell lines expressing either VEGFR1 or VEGR2 (Table 4). Moreover, VEGF Trap was highly effective at reducing VEGF-A-induced calcium signaling in HUVEC, where it was ~130-fold more potent than ranibizumab (Table 3). In addition to promoting endothelial cell proliferation and vascular permeability, VEGF-A is powerful mediator of endothelial cell migration [25]. Consistent with the high potency of VEGF Trap to neutralize VEGF receptor activation, VEGF Trap was highly effective at blocking HUVEC migration induced by VEGF-A_165_. In agreement with previous reports [38, 54], ranibizumab and bevacizumab were also effective at decreasing HUVEC migration, though they were less potent than VEGF Trap, such that a 10- to 100-fold molar excess of ranibizumab or bevacizumab was required to completely block VEGF-induced HUVEC migration, while VEGF Trap was effective at equimolar concentrations.

In the present studies, the ability of ranibizumab to neutralize VEGF-A activity in cell-based assays was only moderately better than that of bevacizumab. For example, the IC_50_ values for inhibition of activation of VEGFR1 and VEGFR2 by 20 pM VEGF-A were less than twofold lower for ranibizumab than bevacizumab (Table 3). This corresponded closely to the observed differences in the binding kinetics of ranibizumab and the full length bivalent bevacizumab antibody, where the *K*_D_ of bevacizumab for VEGF-A was within twofold of that of ranibizumab, as determined by both Biacore and KinExA assays (Tables 1, 2, 4). Interestingly, bevacizumab was ~fivefold more potent than ranibizumab at neutralizing VEGF-A induced calcium influx in HUVEC. This finding may reflect the ~threefold faster association rate of bevacizumab (Table 1), as *k*_a_ is a critical determinant of potency in relatively acute cell-based assays.

The above findings stand in contrast to those recently described by Yu et al. [40]. Specifically, ranibizumab and VEGF Trap were reported to be equally effective in blocking endothelial cell proliferation and migration in HUVEC, while bevacizumab was approximately tenfold less potent. Evaluation of MAPK phosphorylation, which reflects activation of intracellular signaling pathways downstream of the VEGF receptors, showed that all three agents completely blocked MAPK phosphorylation when the VEGF inhibitors were pre-incubated with VEGF-A overnight, before addition to the cells, while VEGF Trap was more potent than either ranibizumab or bevacizumab when preincubated with VEGF-A for shorter time periods (5 and 30 min). The apparent discrepancies with findings of the present study are likely attributable to the fact that Yu et al. [40] utilized higher concentrations of exogenous VEGF-A in all of their cell-based assays, in the range of 0.15–1.25 nM. In other words, the concentration of ligand was above the *K*_D_ values for ranibizumab and bevacizumab, as well as VEGF Trap (Table 1); under these assay conditions the IC_50_ is determined primarily by the concentration of ligand relative to that of the blocker, rather than by the binding affinity. Therefore, precise evaluation of the relative activity of different inhibitors in bioassays requires utilization of the lowest amount of VEGF-A practicable, so that the IC_50_ can reflect differences in binding affinity and not simply inhibition of activity at stoichiometric concentrations of inhibitor, which predominates under conditions where both antibody and ligand concentrations are well above the *K*_D_.

For example, several studies published to date have reported that ranibizumab and bevacizumab are equally effective in neutralizing VEGF-induced endothelial cell proliferation at ‘clinically relevant’ concentrations, i.e., those that obtain in the eye shortly following intravitreal injection [38, 55], which are well above the equilibrium dissociation constants for both antibodies. Differences in activity emerge only when lower concentrations of drug are evaluated, or where acute bioassay readouts reflect differences in association rate constants. For example, Klettner et al. [39], reported that at lower concentrations ranibizumab more efficiently neutralized VEGF secreted from retinal-choroidal cultures than did bevacizumab. Costa et al. [54] also reported that ranibizumab was moderately more effective at inhibiting endothelial cell proliferation than bevacizumab, while in an acute assay bevacizumab more effectively inhibited VEGF-stimulated VEGFR2 and MAPK phosphorylation in human microvascular endothelial cells.

Binding kinetics and affinity are key determinants of the biological activity of antibody-like drugs. In addition to binding affinity, the activity of a drug is also influenced by the concentration present at the site of target activity, which is in turn dependent on tissue distribution and clearance, with larger molecules typically having longer half-lives. With respect to ocular delivery, it was estimated that biologically active concentrations of ranibizumab would be maintained in the vitreous for approximately 4 weeks following intravitreal injections of 0.5 mg [26, 56]. Indeed, monthly injection of 0.5 mg ranibizumab has proven to be the most effective regimen for the treatment of neovascular AMD, based on the outcomes of several phase III clinical trials [29, 57–60], and is the currently approved regimen for treating this disease. Using mathematical modeling, and the then available information on intravitreal clearance and binding affinities, Stewart [61] predicted that the anti-VEGF bioactivity present in the vitreous 30 days following intravitreal (IVT) injection of 0.5 mg ranibizumab would be equivalent to that present at 27–38 days following an injection of 1.25 mg bevacizumab. More recently, using the same modeling approach, Stewart and Rosenfeld [62] predicted the intraocular biological activity comparable to that of 0.5 mg ranibizumab at 30 days post-injection would be maintained for approximately twice that time following injection of 0.5 mg VEGF Trap, and potentially as long as 12 weeks following IVT injection of 2 mg VEGF Trap. This substantial theoretical increase in the relative duration of VEGF neutralizing activity was driven primarily by the higher binding affinity of VEGF Trap for VEGF-A compared to ranibizumab, with a lesser contribution of the predicted longer intravitreal half-life of VEGF Trap (e.g. 4.7 days in rabbits, compared to ~2.9 days for ranibizumab, [63, 64]. Thus, modeling studies suggested that intravitreal administration of the current clinical doses of ranibizumab and bevacizumab would result in effective VEGF-A inhibition of relatively similar duration, while VEGF Trap might be as efficacious as ranibizumab, but with less frequent dosing.

While it remains to be unequivocally determined whether the durations of bioactivity of these VEGF blockers predicted by the above modeling studies will be confirmed by clinical experience, data available to date suggest that the results of these modeling studies may prove reasonably accurate. For example, several clinical studies have investigated alternative strategies to monthly ranibizumab injection, including quarterly (every 3 months) or pro renata (PRN) injections following a treatment initiation phase comprising 3 monthly loading doses. Most large, well-controlled studies conducted to date have found that improvements in visual acuity attained during the initiation phase are lost during the quarterly or PRN maintenance phases [58–60, 65]. The recent CATT Trial produced the best results obtained to date using PRN dosing of ranibizumab, which was statistically non-inferior to that of monthly ranibizumab. This may reflect the fact that in the CATT study patients were followed monthly and rigorous criteria were established for retreatment [32]. Nevertheless, the mean improvement in visual acuity attained in CATT using PRN ranibizumab was 1.6 letters below that of monthly ranibizumab, at the end of 1 year. Importantly, the effect of bevacizumab given monthly on visual outcomes was within 0.4 letters of that obtained with ranibizumab given monthly. However, bevacizumab administered PRN failed non-inferiority comparisons to monthly regimens for both antibodies, despite the fact that it was administered more frequently than ranibizumab PRN. These findings are in line with the predictions of modeling studies, as well as the results of the present report, which indicate that the binding affinity and in vitro activity of bevacizumab are moderately less than those of ranibizumab. Several additional large scale controlled trials are currently in progress to evaluate the effects of these two antibodies in patients with neovascular AMD, using both fixed and PRN dosing schedules [31]. These studies, together with outcomes from the CATT trial following longer-term treatment, should provide a clearer picture of the relative clinical activity, and safety, of ranibizumab and bevacizumab.

Although fewer clinical trials have been conducted to date with VEGF Trap-Eye, the available data suggest that, as predicted in modeling studies, the increased affinity of VEGF Trap for VEGF-A may be reflected in clinical activity. For example, in a recent double masked phase 2 trial (CLEAR-IT 2) patients with exudative AMD were randomized to an initiation phase of either a single, or monthly IVT injections of VEGF Trap for 12 weeks at doses of either 0.5 or 2 mg. Patients were then switched to a PRN regimen at their originally assigned doses. Reports of the 1 year results described maintenance of statistically significant improvements in vision, retinal thickness and size of the CNV lesions [66, 67]. Here, patients initially dosed on a 2.0 mg monthly schedule received, on average, only 1.6 additional injections during the 40 week PRN period, and those initially dosed on a 0.5 mg monthly schedule received, on average, 2.5 injections. More recently, 1 year results have been reported from two phase 3 clinical trials (VIEW 1 and VIEW 2) in which VEGF Trap-Eye was dosed monthly at 0.5 or 2.0 mg in patients with wet AMD, or at 2.0 mg every other month following an initiation phase of 3 monthly doses. All VEGF Trap-Eye treatment arms, including the 2.0 mg every other month treatment regimen, produced improvements in visual acuity that were equivalent to that obtained in patients dosed with 0.5 mg ranibizumab monthly [68, 69].

The development of ranibizumab has demonstrated that binding multiple VEGF-A isoforms is of substantial benefit in the treatment of neovascular AMD, compared to treatment with pegaptanib, which binds only the 165 isoform of VEGF-A [23, 29, 57, 70–72]. Recent studies have implicated additional VEGF family members, notably PlGF and VEGF-B, in the pathology of ocular vascular diseases as well as some cancers [8, 16, 73]. Therefore, a unique potential advantage of VEGF Trap relative to ranibizumab and bevacizumab is that it also binds VEGF-B and PlGF with high affinity. PlGF in particular has been shown to act in concert with VEGF-A to promote pathological angiogenesis, vascular leak and inflammation [8, 11, 18, 74], and like VEGF-A, levels of PlGF are elevated in the eyes of patients with diverse ocular vascular diseases, including wet AMD [15, 75]. Furthermore, genetic deletion or pharmacological inhibition of PlGF has been shown to inhibit choroidal neovascularization and inflammation, and to enhance the activity of VEGF-A targeted molecules in animal models of choroidal neovascularization [13, 16]. More recently, it has been reported that overexpression of VEGF-B in the murine retina, via adeno-associated virus gene transfer, also promotes retinal and choroidal neovascularization and blood-retinal barrier breakdown [76]. These studies suggest that targeting PlGF and VEGF-B, in addition to VEGF-A, could be of added benefit in treating angiogenic ocular disorders.

Similarly, targeting these additional factors may be important in the oncology setting. First, these VEGF family ligands, most notably PlGF, have been implicated in promoting tumor growth [8, 16, 73], therefore inhibiting these factors, in addition to VEGF-A, may prove therapeutically beneficial in treating cancer. Bevacizumab, which inhibits only VEGF-A, is approved for use in various cancer treatment settings. VEGF Trap, while not currently approved for use, has also exhibited efficacy in the oncology setting. Most recently it was reported to have an overall survival benefit in metastatic colorectal cancer [77]. Changes in the levels of PlGF and other factors have been observed in patients with metastatic colorectal cancer treated with bevacizumab, during and following cessation of treatment [78, 79], and the authors of both studies suggested that increases in other pro-angiogenic factors may be one mechanism underlying the development of resistance to anti-VEGF therapy. However, further prospective evaluations are needed to confirm these hypotheses.

In summary, VEGF Trap demonstrated higher binding affinity for VEGF-A isoforms and greater potency in vitro than ranibizumab or bevacizumab. These attributes, in addition to its ability to bind VEGF-B and PlGF, could be of added benefit in treating various ocular disorders and cancers.

## Electronic supplementary material

Below is the link to the electronic supplementary material.

Supplementary material 1 (PDF 1284 kb)

## Abbreviations

AMD: Age-related macular degeneration
HUVEC: Human umbilical vein endothelial cells
VEGF: Vascular endothelial growth factor
VEGFR: VEGF receptor
PlGF: Placental growth factor
