# Supplementary material for: Binding and neutralization of vascular endothelial growth factor (VEGF) and related ligands by VEGF Trap, ranibizumab and bevacizumab
Source: Angiogenesis. 2012 Feb 3;15(2):171–85. doi: 10.1007/s10456-011-9249-6 (PMC3338918; doi:10.1007/s10456-011-9249-6)
Supplement: Supplementary file 1 — Supplementary material 1 (PDF 1284 kb) [file 10456_2011_9249_MOESM1_ESM.pdf]

Article Title: Binding and Neutralization of Vascular Endothelial Growth Factor (VEGF) and Related Ligands by VEGF Trap, Ranibizumab and Bevacizumab

Authors: Nicholas Papadopoulos, Joel Martin, Qin Ruan, Ashique Rafique, Michael P. Rosconi, Ergang Shi, Erica A. Pyles, George D. Yancopoulos, Neil Stahl and Stanley J. Wiegand

To whom correspondence should be addressed: Stanley J. Wiegand, Regeneron Pharmaceuticals Inc., 777 Old Saw Mill River Road, Tarrytown, NY 10591, USA, Tel.: (914) 345-7740; Fax: (914) 347-5045; Email: stanley.wiegand@regeneron.com

Journal: Angiogenesis

Online Resource 1:

Figure Legends:

Fig. 1 Kinetic analyses of VEGF Trap, bevacizumab, and ranibizumab binding to VEGF-A_{165}. VEGF Trap (47 RU) and bevacizumab (67 RU) were captured on a Protein A coupled chip surface, while ranibizumab (27 RU) was captured on an anti-human Fab coupled chip surface. Human VEGF-A_{165} was tested in duplicate in a 2-fold dilution series, the association phase of VEGF-A_{165} ligand was monitored at 100 µl/min for 2-3 minutes over each of the captured surfaces. Representative sensorgrams of **A**) VEGF Trap binding to VEGF-A_{165}, 1, 0.5, 0.25, 0.125, and 0.0625 nM, **B**) bevacizumab binding to VEGF-A_{165}, 100, 50, 25, 12.5, 6.25, and 3.125 nM, and **C**) ranibizumab binding to VEGF-A_{165}, 100, 50, 25, 12.5, and 6.25 nM, are shown as black lines. The data were globally fit to a 1:1 binding with mass-transfer interaction model with a fixed k_d (determined by monitoring dissociation for up to 3 hours) using BiaEvaluation 4.1. Kinetic fits from the analyses are overlaid on the binding data in red.

Fig. 2 Kinetic analyses of VEGF Trap, bevacizumab, and ranibizumab binding to human PlGF-2. VEGF Trap (71 RU) and bevacizumab (147 RU) were captured on a Protein A coupled chip surface, while ranibizumab (154 RU) was captured on an anti-human Fab coupled chip surface. Human PlGF-2 was tested in duplicate in a 2-fold dilution series, the association phase of PlGF ligands was monitored at 50 µl/min for 5 minutes over each of the captured surfaces. Representative sensorgrams of **A**) VEGF Trap binding to human PlGF-2, 5, 2.5, 1.25, 0.625, 0.3125, 0.156, and 0.078 nM, **B**) bevacizumab binding to human PlGF-2, 10, 5, 2.5, 1.25, 0.625, 0.3125, 0.156, and 0.078 nM, and **C**) ranibizumab binding to human PlGF-2, 10, 5, 2.5, 1.25, 0.625, 0.3125, 0.156, and 0.078 nM, are shown as black lines. The data were globally fit to a 1:1 binding with mass-transfer interaction model using BiaEvaluation 4.1. Kinetic fits from the analyses are overlaid on the binding data in red.

Fig. 3 Kinetic analyses of VEGF Trap binding to various VEGF related ligands. VEGF Trap (43-57 RU) was captured (on a Protein A coupled chip surface) and various concentrations of VEGF related ligands were tested in duplicate in a 2-fold dilution series. The association phase of VEGF related ligands was monitored at 100 µl/min for 2 minutes over each of the captured surfaces. Representative sensorgrams of VEGF Trap binding to **A**) hVEGF-A_{121}, 1, 0.5, 0.25, 0.125, and 0.0625 nM, **B**) mVEGF-A_{120}, 2, 1, 0.5, 0.25, 0.125, and 0.0625 nM, **C**) mVEGF-A_{164}, 1, 0.5, 0.25, 0.125, and 0.0625 nM, **D**) rVEGF-A_{164}, 2, 1, 0.5, 0.25, 0.125, and 0.0625 nM, and **E**) rabbit VEGF-A_{165}, 2, 1, 0.5, 0.25, 0.125, and 0.0625 nM, are shown as black lines. The data were globally fit to a 1:1 binding with mass-transfer interaction model with a fixed k_d (determined by monitoring dissociation for up to 3 hours) using BiaEvaluation 4.1. Kinetic fits from the analyses are overlaid on the binding data in red.

Fig. 4 Kinetic analyses of VEGF Trap binding to human PlGF-1, mouse PlGF-2 and human VEGF-$B_{(10\text{-}108)}$. **A & B)** VEGF Trap (69-270 RU) was captured on a Protein A coupled chip surface and various concentrations of human PlGF-1 and mouse PlGF-2 were tested in duplicate in a 2-fold dilution series. The association phase of PlGF ligands was monitored at 50 µl/min for 5 minutes over each of the captured surfaces. Representative sensorgrams of VEGF Trap binding to **A**) human PlGF-1, 10, 5, 2.5, 1.25, 0.625, 0.3125 nM, and **B**) mouse PlGF-2, 1.25, 0.625, 0.3125, 0.156, and 0.078 nM, are shown as black lines. **C)** VEGF Trap (76 RU) was captured on a Protein A coupled chip surface and various concentrations of human VEGF-$B_{(10\text{-}108)}$ were tested in duplicate in a 2-fold dilution series. The

association phase of VEGF-$B_{(10\text{-}108)}$ ligand was monitored at 100 µl/min for 2 minutes over the captured surface. Representative sensorgrams of VEGF Trap binding to human VEGF-$B_{(10\text{-}108)}$, 2.5, 1.25, 0.625, 0.3125, and 0.156 nM are shown as black lines. The data were globally fit to a 1:1 binding with mass-transfer interaction model using BiaEvaluation 4.1. Kinetic fits from the analyses are overlaid on the binding data in red.

Fig. 5 Kinetic analyses of **A**) hVEGFR1-hFc and **B**) hVEGFR2-hFc, binding to human VEGF-A_{165}. hVEGFR1-hFc (106 RU) or hVEGFR2-hFc (127 RU) was captured on a Protein A coupled chip surface and various concentrations of human VEGF-A_{165} were tested in duplicate in a 2-fold dilution series. The association phase of human VEGF-A_{165} was monitored at 100 µl/min for 90 seconds over each of the captured surfaces. Representative sensorgrams **A**) hVEGFR1-hFc binding to VEGF-A_{165}, 1.25, 0.625, 0.3125, and 0.156 nM, and **B**) hVEGFR2-hFc binding to VEGF-A_{165}, 5, 2.5, 1.25, 0.625, and 0.3125 nM are shown as black lines. The data were globally fit to a 1:1 binding with mass-transfer interaction model. Kinetic fits from the analyses (red) are overlaid on the binding data in black. **C)** Binding of human VEGF-C and human VEGF-D to VEGF Trap, hVEGFR1-hFc and hVEGFR3-hFc. VEGF Trap, hVEGFR1-hFc and VEGFR3-hFc were captured on Protein A sensor surfaces at 420 RU, 294 RU and 316 RU, respectively. A concentration of 100 nM of each ligand (hVEGF-A_{165}, hVEGF-C and hVEGF-D) was injected over the captured receptor surfaces at a flow rate of 50 µl/min for 5 minutes. The specific binding RU of each ligand to each captured receptor surface was normalized according to femtomole level of each receptor captured on the Protein A coupled surface. Note: The "RU/fmole receptor captured" refers to the RU value of the bound ligand per femtomole of receptor captured per mm^2 sensor. The femtomole of each captured receptor/mm^2 was calculated based on 1000 RU=1ng/mm^2 (Handbook of Biochemistry and Molecular biology, 3rd ed. Vol II, CRC Press, Cleveland (1976).

Fig. 6 A) Solution equilibrium analysis of the VEGF-A_{165}/VEGF Trap interaction. KinExA n-curve analysis. Samples of VEGF Trap, at starting concentrations of 5 (circle), 2.5 (box) or 1 (triangle) pM, were mixed with human VEGF-A_{165} and allowed to reach equilibrium. The time to reach equilibrium ranged from 10 to 36 hours depending on the initial concentration of VEGF Trap. The concentration of hVEGF-A_{165} or VEGF-A_{121} ranged between 100 pM and 97.7 fM (5 pM VEGF Trap), 50 and 48.8 fM (2.5 pM VEGF Trap) and 20 and 19.5 fM (1 pM VEGF Trap). Azlactone beads with coupled hVEGF-A_{165} were used to measure the concentration of free VEGF Trap at equilibrium. VEGF Trap bound to the beads was quantified with Cy-5 labeled anti-human Fc polyclonal antibody. **B)** 95% confidence interval error graphs for VEGF-A_{165}/VEGF Trap interaction. **C**) Solution equilibrium analysis of the VEGF-A_{121}/VEGF Trap interaction. Samples of VEGF Trap, at starting concentrations of 5 (box), 2.5 (triangle) or 1 (circle) pM, were mixed with VEGF-A_{121} and allowed to reach equilibrium. Experimental conditions were identical to those used for the VEGF-A_{165}/VEGF Trap interaction. **D**) 95% confidence interval error graphs for VEGF-A_{121}/VEGF Trap interaction. **E)** Solution equilibrium analysis of the PLGF-2/VEGF Trap interaction. Samples of VEGF Trap, at a starting concentration of 50 pM, were mixed with human PLGF-2 and allowed to reach equilibrium. The time to reach equilibrium was 18 hours. The concentration of PLGF-2 ranged between 5 nM and 0.5 pM. **F)** 95% confidence interval error graph for the PLGF-2/VEGF Trap interaction. **G**) Solution equilibrium analysis of the VEGF-$B_{(10\text{-}108)}$/VEGF Trap interaction. Samples of VEGF Trap at a starting concentration of 15 pM, were mixed with human VEGF-$B_{(10\text{-}108)}$ and allowed to reach equilibrium. The time to reach equilibrium was 18 hours. The concentration of VEGF-$B_{(10\text{-}108)}$ ranged between 1.25nM and 610 fM. Azlactone beads with coupled hVEGF-A_{165} were used to measure concentration of free VEGF Trap at equilibrium. **H)** 95% confidence interval error graph for the VEGF-$B_{(10\text{-}108)}$/VEGF Trap interaction.

Fig. 7 Solution equilibrium analysis of the VEGF-A_{165}/ranibizumab interaction and the VEGF-A_{165}/bevacizumab interaction. KinExA n-curve analysis. **A**) Samples of ranibizumab, at a starting concentration of 50 (circle), 150 (triangle) or 400 (box) pM, were mixed with human hVEGF-A_{165} and allowed to reach equilibrium. The time to reach equilibrium ranged from 72 to 96 hours depending on the initial concentration of ranibizumab. The concentration of hVEGF-A_{165} ranged between 15 nM and 14.6 pM (400 pM ranibizumab), 3 nM and 2.93 pM (150 pM ranibizumab) and 750 pM and 0.73 pM (50 pM ranibizumab). **C**) Samples of bevacizumab, at a starting concentration of 25 (circle), or 50 (box) pM, were mixed with human hVEGF-A_{165} and allowed to reach equilibrium. The time to reach equilibrium was 72 hours. The concentration of hVEGF-A_{165} ranged between 5 nM and 2.4 pM (50 pM bevacizumab) and 1 nM and 0.49 pM (25 pM bevacizumab). Azlactone beads with coupled hVEGF-A_{165} were used to measure the concentration of free ranibizumab or bevacizumab at equilibrium. Ranibizumab or bevacizumab bound to the beads was quantified with Cy-5 labeled anti-human kappa polyclonal antibody. **B & D**) 95% confidence interval error graphs for the VEGF-A_{165}/ranibizumab interaction and the VEGF-A_{165}/bevacizumab interaction, respectively.

Table 1 Summary of kinetic binding parameters for VEGF Trap binding to VEGF family related ligands from multiple species

**Ligand**	**Kinetic Binding Parameters**		
	$k_a/10^5$ **(M^{-1}s^{-1})**	$k_d/10^{-5}$ **(s^{-1})**	K_D **(pM)**
Mouse VEGF-A_{120}	215.0 (4.0)	1.23 (0.01)	0.572
Mouse VEGF-A_{164}	280.0 (7.0)	1.64 (0.006)	0.586
Rat VEGF-A_{164}	367.0 (6.0)	1.73 (0.006)	0.471
Rabbit VEGF-A_{165}	339.0 (6.0)	2.63 (0.02)	0.776
Mouse PlGF-2	164.0 (0.2)	5.45 (0.03)	3.32
Human PlGF-1	67.3 (0.2)	264 (1.0)	392
Human VEGF-C	NB	NB	NB
Human VEGF-D	NB	NB	NB

NB: No binding observed under the assay conditions used.
VEGF Trap was captured via a Protein A-coupled sensor chip.
Numbers in parentheses represent the standard error of the kinetic fit.

Fig. 1

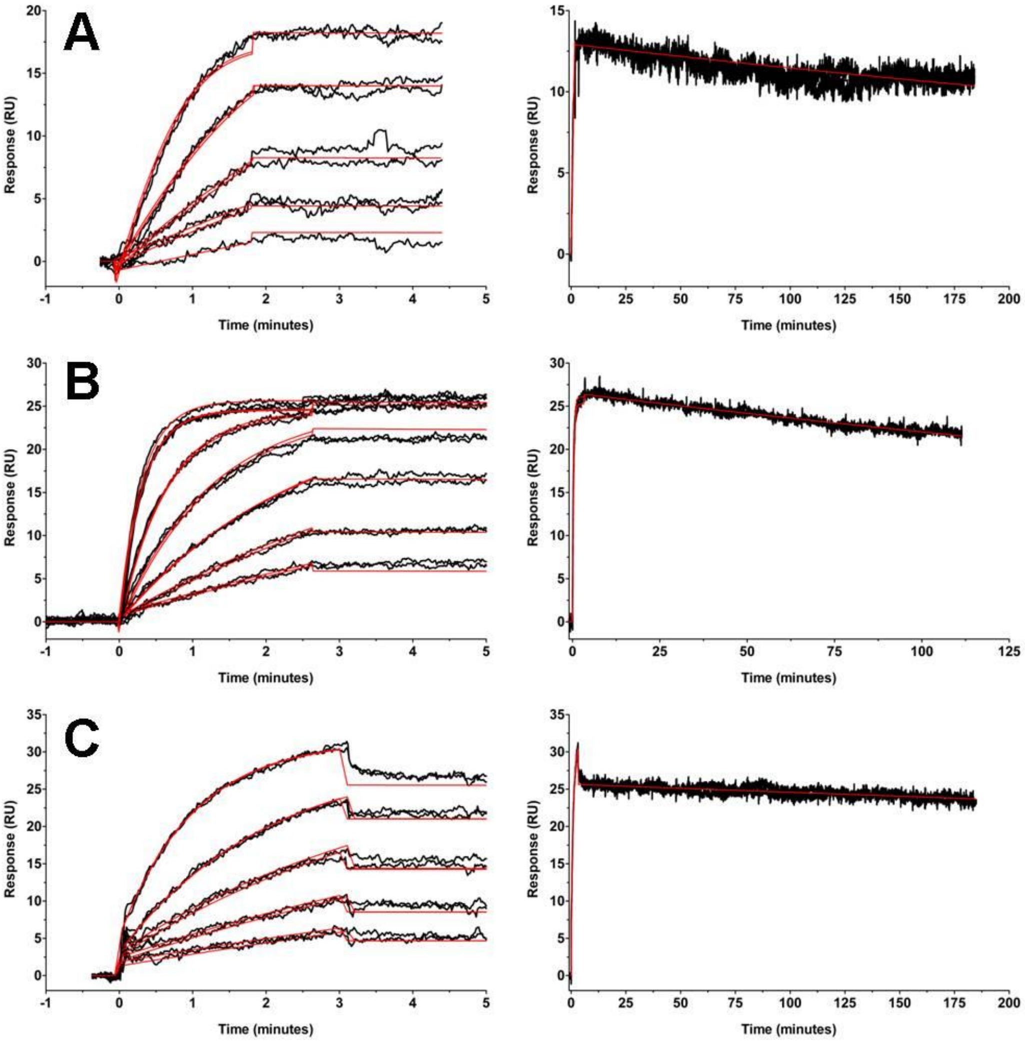

Fig. 2

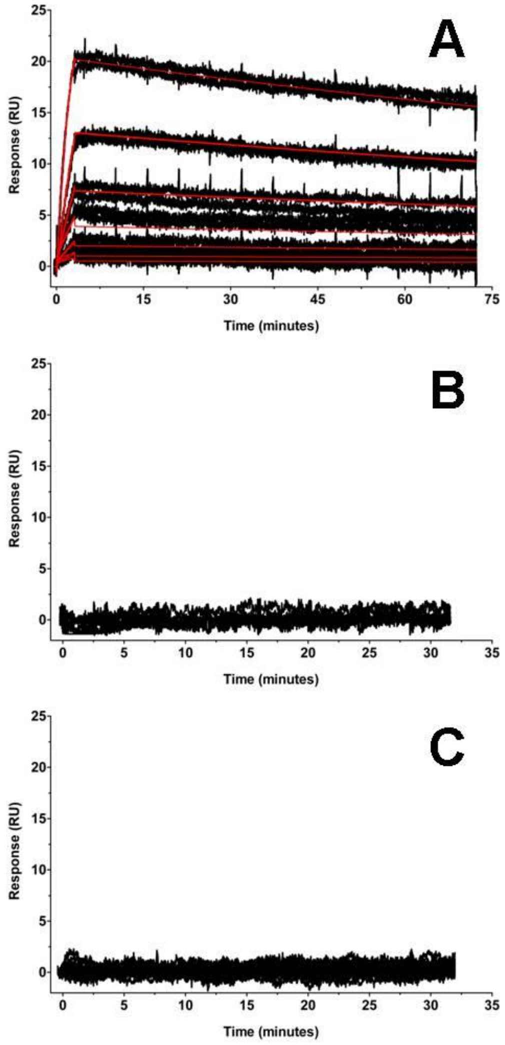

Fig. 3

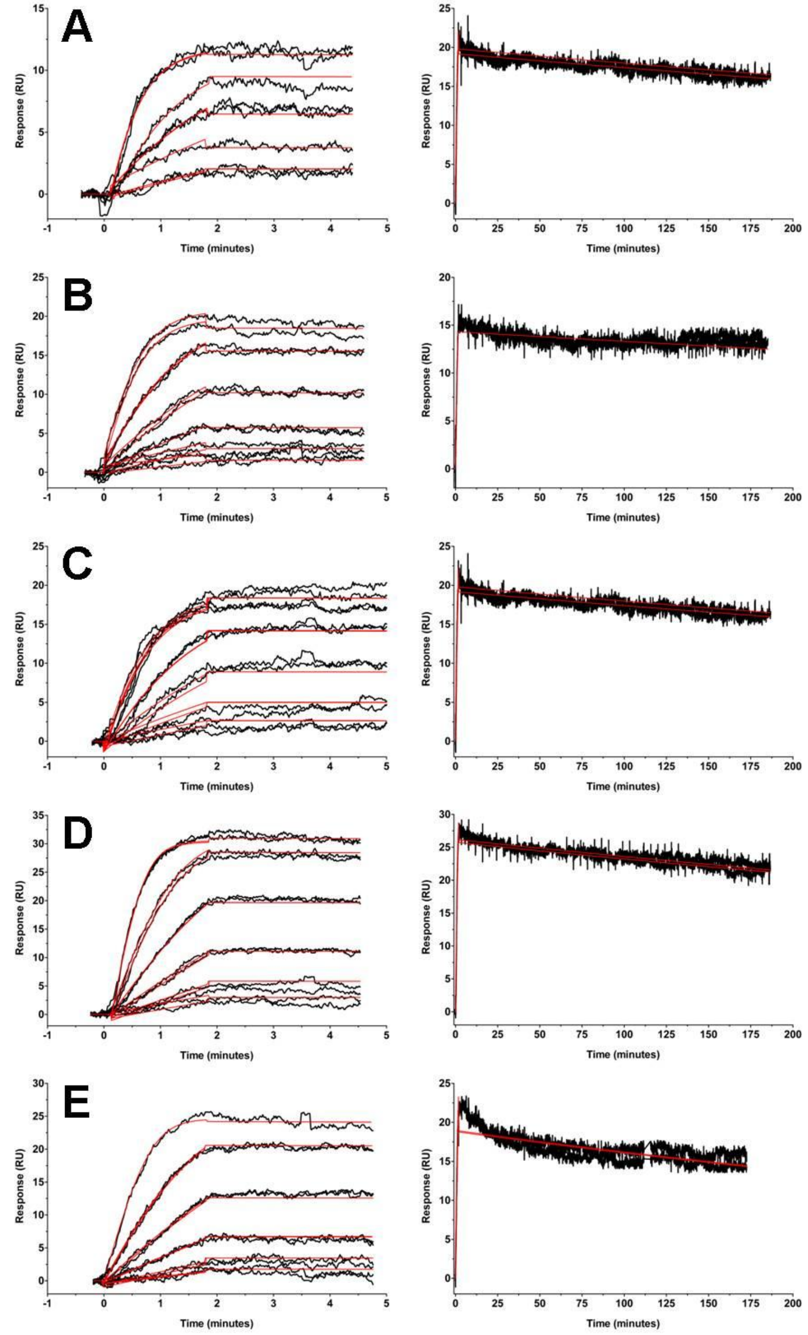

Fig. 4

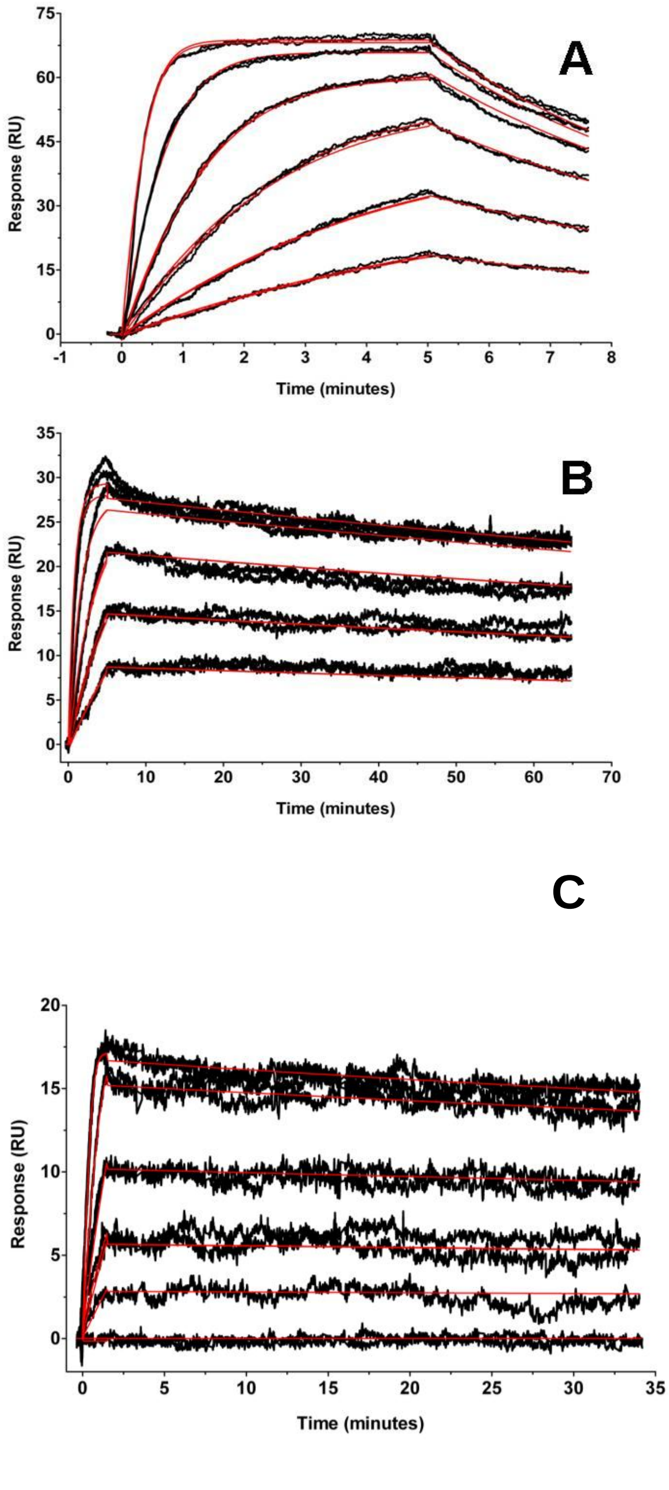
A
Response (RU)
75
60
45
30
15
0
-1
0
1
2
3
4
5
6
7
8
Time (minutes)
B
Response (RU)
35
30
25
20
15
10
5
0
0
10
20
30
40
50
60
70
Time (minutes)
C
Response (RU)
20
15
10
5
0
0
5
10
15
20
25
30
35
Time (minutes)

Fig. 5

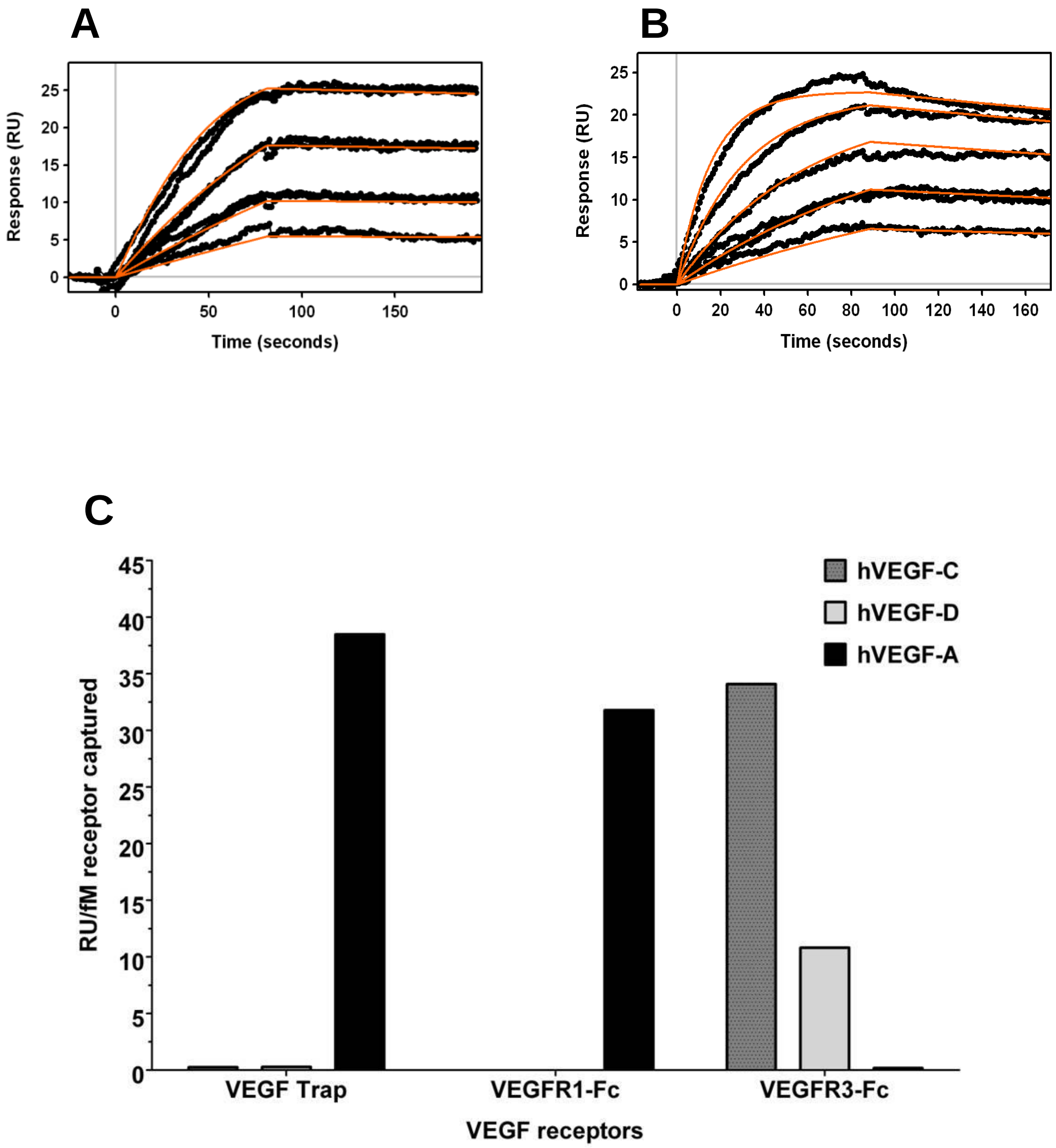

A
Response (RU)
0
5
10
15
20
25
0
50
100
150
Time (seconds)
B
Response (RU)
0
5
10
15
20
25
0
20
40
60
80
100
120
140
160
Time (seconds)
C
RU/fM receptor captured
0
5
10
15
20
25
30
35
40
45
hVEGF-C
hVEGF-D
hVEGF-A
VEGF Trap
VEGFR1-Fc
VEGFR3-Fc
VEGF receptors

Fig. 6

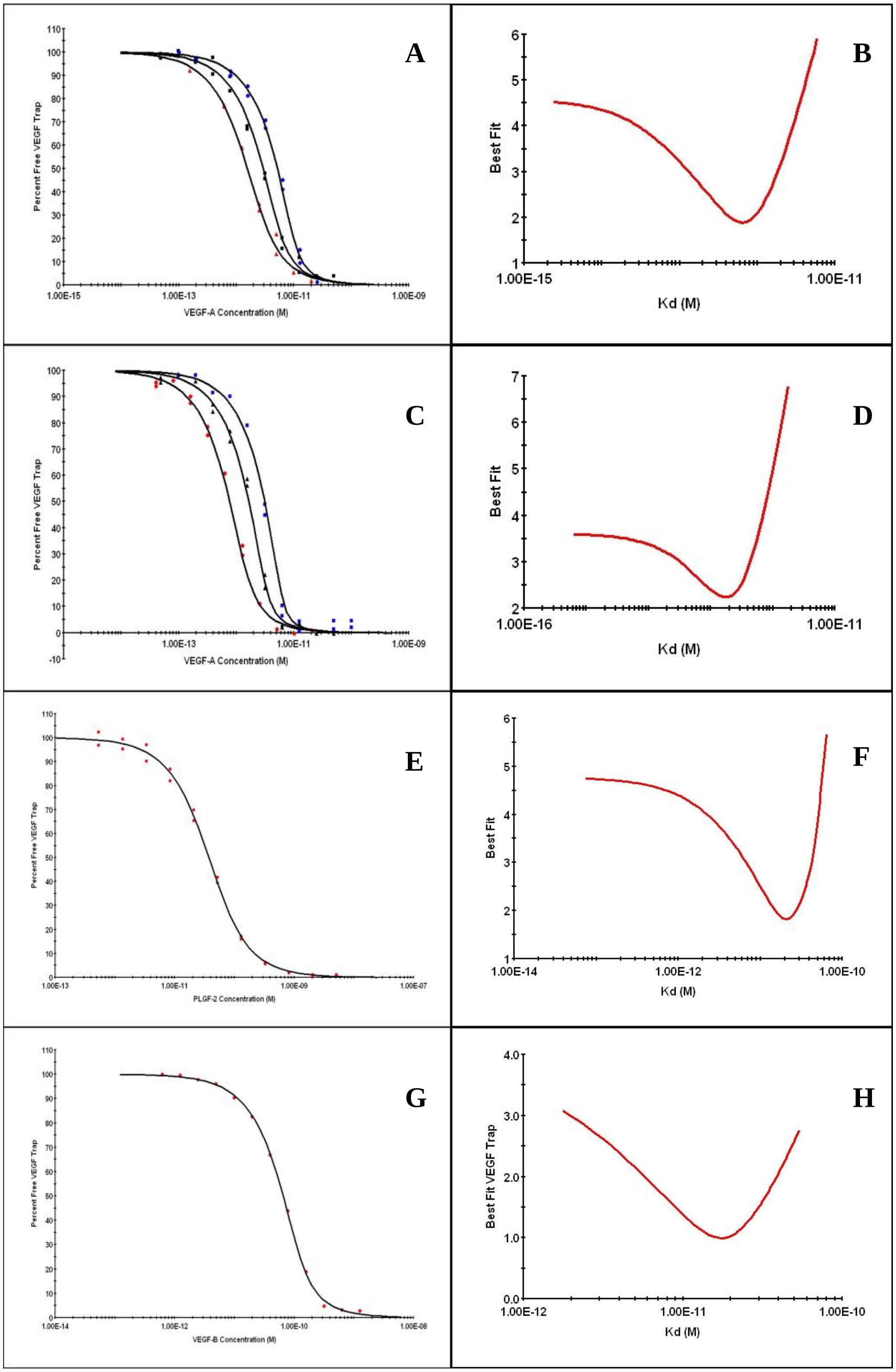
A
Percent Free VEGF Trap
VEGF-A Concentration (M)
1.00E-15
1.00E-13
1.00E-11
1.00E-09
B
Best Fit
Kd (M)
1.00E-15
1.00E-11
C
Percent Free VEGF Trap
VEGF-A Concentration (M)
1.00E-13
1.00E-11
1.00E-09
D
Best Fit
Kd (M)
1.00E-16
1.00E-11
E
Percent Free VEGF Trap
PLGF-2 Concentration (M)
1.00E-13
1.00E-11
1.00E-09
1.00E-07
F
Best Fit
Kd (M)
1.00E-14
1.00E-12
1.00E-10
G
Percent Free VEGF Trap
VEGF-B Concentration (M)
1.00E-14
1.00E-12
1.00E-10
1.00E-08
H
Best Fit VEGF Trap
Kd (M)
1.00E-12
1.00E-11
1.00E-10

Fig. 7

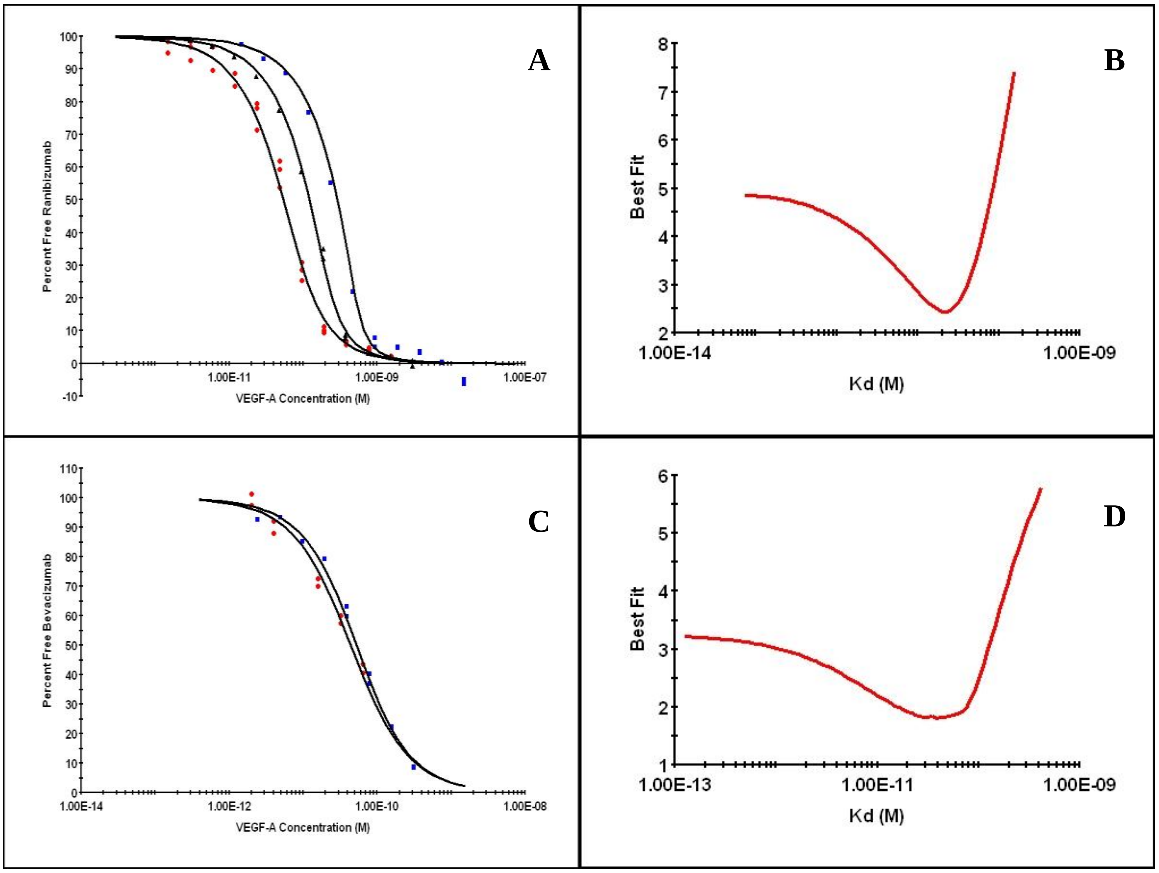
A
Percent Free Ranibizumab
VEGF-A Concentration (M)
1.00E-11
1.00E-09
1.00E-07
B
Best Fit
Kd (M)
1.00E-14
1.00E-09
C
Percent Free Bevacizumab
VEGF-A Concentration (M)
1.00E-14
1.00E-12
1.00E-10
1.00E-08
D
Best Fit
Kd (M)
1.00E-13
1.00E-11
1.00E-09
